# Pattern and prognostic value of cardiac involvement in patients with late-onset pompe disease: a comprehensive cardiovascular magnetic resonance approach

**DOI:** 10.1186/s12968-016-0311-9

**Published:** 2016-12-07

**Authors:** Matthias Boentert, Anca Florian, Bianca Dräger, Peter Young, Ali Yilmaz

**Affiliations:** 1 Department of Sleep Medicine and Neuromuscular Disorders, University Hospital Münster, Münster, Germany; 2 Department of Cardiovascular Medicine, University Hospital Münster, Albert-Schweitzer-Campus 1, building A1, 48149 Münster, Germany

**Keywords:** Pompe disease, Cardiac disease, Cardiomyopathy, Cardiovascular magnetic resonance, Late gadolinium enhancement, Feature tracking, Mapping

## Abstract

**Background:**

Pompe disease is an autosomal recessive disorder caused by deficiency of the lysosomal α–1,4-glucosidase leading to accumulation of glycogen in target tissues with progressive organ failure. While the early infantile-onset form is characterized by early severe hypertrophic cardiomyopathy with cardiac and respiratory failure, clinically relevant cardiomyopathy seems to be uncommon in patients with late-onset Pompe disease, and the prevalence and nature of myocardial abnormalities are still to be clarified.

**Methods:**

Seventeen patients with genetically proven late-onset Pompe disease (50 ± 18 years, 11 male) and 18 age- and gender-matched healthy controls (44 ± 10 year, 12 male) underwent comprehensive cardiovascular magnetic resonance (CMR) including conventional and advanced techniques: cine and feature tracking-based strain imaging for depiction of (even subtle) systolic LV dysfunction as well as late gadolinium enhancement (LGE) and myocardial extracellular volume fraction (ECV) quantification for focal and diffuse fibrosis detection.

**Results:**

All patients had normal left ventricular (LV) and right ventricular (RV) volumes and normal LV and RV ejection fraction. In comparison to healthy controls, neither conventional cine nor advanced feature-tracking based-strain imaging could depict any (subclinical) myocardial systolic dysfunction. Three (18%) of the patients had non-ischemic LGE in the basal inferolateral wall and 21% demonstrated elevated global ECV values suggestive of interstitial myocardial fibrosis. Non-specific abnormalities such as left atrial (LA) dilatation were present in two patients, while LV hypertrophy was seen only in one. Two of the three LGE-positive patients were also hypertensive and demonstrated high global ECV values (>30%) in addition to dilated LA. After a median follow-up of 25 (11–29) months, only one cardiovascular event occurred: one of the LGE-positive patients with a high cardiovascular risk profile suffered an acute coronary syndrome.

**Conclusion:**

In contrast to the early infantile-onset form of Pompe disease, mild and rather non-specific cardiac abnormalities can be detected by CMR only in a small proportion of patients with late-onset Pompe disease. The observed structural abnormalities seem to result from an interplay between the storage disease and other comorbidities and they did not affect short-term to mid-term prognosis in adult Pompe patients.

## Background

Glycogen storage disease type II or Pompe disease is an autosomal recessive disorder caused by deficiency of the lysosomal enzyme α–1,4-glucosidase (GAA) leading to accumulation of glycogen resulting in lysosomal dysfunction, autophagy, and progressive tissue damage [1, 2]. Severity varies by age of onset, rate of progression and extent of organ involvement comprising primarily the skeletal and respiratory muscles, but also the human heart [1]. In the early infantile-onset form of Pompe disease, severe (obstructive) cardiomyopathy with cardiac and respiratory failure along with progressive quadriplegia is present very early, and survival without enzyme replacement therapy (ERT) beyond one year is extremely rare [3]. The term late-onset Pompe disease (LOPD) sums up subtypes with late-infantile, childhood, juvenile, or adult onset of disease. Patients with LOPD suffer from a progressive myopathy of the limb-girdle and trunk muscles, and the diaphragm [4]. While clinically relevant cardiomyopathy is uncommon the prevalence and nature of myocardial abnormalities are still to be clarified [5–8].

Cardiovascular magnetic resonance (CMR) is a highly sensitive tool for detection of functional and structural myocardial abnormalities in both ischemic and non-ischemic cardiomyopathies [9]. In addition to depiction of focal myocardial damage and/or fibrosis by conventional late gadolinium enhancement (LGE), advanced equilibrium contrast CMR techniques for quantification of myocardial extracellular volume fraction (ECV) have been developed and validated for quantification of myocardial interstitial fibrosis [10]. Moreover, feature-tracking CMR using the standard cine images acquired in routine protocols is a novel technique that allows quantification of strain to detect even subtle changes in LV function and has been successfully validated against myocardial tagging [11].

The aim of the present study was to look for even subclinical myocardial involvement by means of comprehensive CMR, including conventional and advanced techniques, in a population of patients with LOPD and no previous history of cardiac disease.

## Methods

### Study population

Seventeen patients with known LOPD (50 ± 18 years, 11 male) were prospectively enrolled between July 2013 and March 2016. Diagnosis was genetically confirmed in all patients by detection of two pathogenic mutations of the *GAA* gene. All patients underwent detailed cardiac and neurologic evaluation including comprehensive CMR studies. In patients receiving home ventilatory support due to severe respiratory muscle weakness, CMR was performed using non-invasive ventilation (NIV) during the procedure. Exclusion criteria were inability to lie flat and perform the required breathholds as well as contraindications to CMR or to gadolinium contrast administration. Additionally, 18 age- and gender-matched healthy subjects (44 ± 10 year, 12 male) were included and represented the control group. All patients were periodically followed up in three months intervals. Any cardiovascular event, including heart failure, acute coronary syndromes, detection of arrhythmia, any cardiac hospitalization and cardiac death, was documented.

### CMR

ECG-gated CMR studies were performed on 1.5 T scanners (Aera, Siemens Medical Solutions, Erlangen, Germany and Achieva, Philips, Best, The Netherlands) using commercially available cardiac software, electrocardiographic triggering, and cardiac-dedicated surface coils. Cine-imaging was performed using a steady-state-free-precession (SSFP) sequence in three long-axis slices (four-, three and two-chamber) and a stack of short-axis slices completely covering the LV. LGE imaging was performed using a T1-weighted inversion recovery gradient-echo sequence 10–15 min after intravenous contrast administration (0.10 mmol/kg Magnevist®) in the same imaging planes as the cine-images. For ECV imaging, T1 measurements were made using a Modified Look-Locker inversion recovery (MOLLI) sequence before and 15–20 min after intravenous contrast (following LGE-imaging) as described elsewhere [12].

### CMR analysis

CMR analysis was performed off-line by two experienced readers. Ventricular volumes, ejection fraction and LV mass were derived by contouring endo- and epicardial borders on the short-axis cine images and indexed to body surface area. The papillary muscles were included in the LV cavity. LV hypertrophy was considered present whenever maximal end-diastolic wall thickness was ≥ 13 mm in men and ≥ 12 mm in women. The ratio of LV mass to end-diastolic volume was used as an index of concentric hypertrophy [13]. Left atrial (LA) surface was measured at end-systole in 4-chamber view and when ≥ 29 cm^2^ in men and ≥ 27 cm^2^ in women defined as LA dilatation [14]. LGE presence and pattern were visually assessed on the short-and long-axis images by using the AHA 17-segment model. LGE pattern was globally assessed as: ischemic (subendocardial and/or transmural) and non-ischemic (subepicardial and/or intramural) [15].

### ECV measurement

For myocardial ECV calculation, motion corrected pre- and post-contrast T1 pixel maps, automatically generated by the scanner after each MOLLI acquisition, were used for processing with Image J (http://rsbweb.nih.gov/ij/) software as previously described [12, 16]. R1 maps were generated by taking the reciprocal of the each T1 map on a pixel-by-pixel basis and ΔR1 maps were computed by subtracting the pre-contrast R1 map from the post-contrast R1 map. For each patient/healthy subject one mid-ventricular short-axis slice served for analysis. Average pixel signal intensity (ΔR1 value) was measured in the chosen short-axis ΔR1 map using two manually delineated regions of interest, one in the septum and one in the lateral wall. Additionally, a region of interest was placed in the blood pool, carefully avoiding papillary muscles and LV trabeculations. The following formula was used for myocardial ECV computation: Extracellular volume fraction (ECV) = λ x (1- hematocrit), where the partition coefficient λ = ΔR1 (myocardium)/ΔR1(blood) (Ref). Global myocardial ECV was obtained by averaging septal and lateral wall ECV values in each patient/control.

### Feature tracking analysis

The feature tracking analysis was performed on the standard acquired cine CMR images using dedicated software (CVI42, Circle Imaging, Leiden, Netherlands). Firstly, landmarks for LV base (at the mitral valve ring) and apex were defined in all three long-axis slices at end-diastole. Following, endocardial and epicardial borders were manually delineated in the end-diastolic frame in the three long-axis slices and in the short-axis stack, from the most basal slice without trough plane distortion from the LV outflow tract to the most apical one. Both the landmarks and contours were automatically propagated through the cardiac cycle and manually corrected in case of inaccuracies. Global peak systolic longitudinal strain (GLS) was derived from the long-axis cines, while short-axis cines were used to derive global peak circumferential (GCS) and radial strains (GRS) [11].

### Statistical analysis

Continuous variables with normal distribution are expressed as mean and standard deviation (SD). Skewed variables were expressed as median and interquartile range (IQR). Categorical variables are expressed as frequency with percentage. t-Student test was used for comparison of normally distributed characteristics between patients and controls. Levene’s test was used for testing equality of variances. The chi-square test with Yate’s correction was used to compare non-continuous variables expressed as proportions. Statistical analysis was performed using SPSS software for Windows (version 19.0, IBM Corp., Armonk, NY). A *p*-value ≤ 0.05 was considered statistically significant.

## Results

### General characteristics of the study population

General characteristics of patients and healthy controls are shown in Table 1. Seventeen patients with LOPD (juvenile vs. adult disease onset: 24 vs. 76%) had a mean age of 50 ± 18 years (median and IQR: 30 year; 20–43 years) and 65% were male (*n* = 11). Six patients with LOPD (35%) had known hypercholesterolemia, significantly more frequent compared to controls. Additionally, three patients (18%) had arterial hypertension, all under pharmacological treatment, and two patients had diabetes in comparison to none of the controls. None of the patients with LOPD had known cardiac disease at study inclusion.

**Table 1:** General characteristics of the patients and controls

	Pompe	Controls	*P*-Value
	*N* = 17	*N* = 18	
Age, years	50 ± 18	44 ± 10	0.24
Male, *n* (%)	11 (65)	12 (67)	1.00
BMI, kg/m^2^	24 ± 4	24 ± 3	0.95
Hypertension, *n* (%)	3 (18)	0 (0)	0.10
Diabetes, *n* (%)	2 (12)	0 (0)	0.23
Hypercholesterolemia, *n* (%)	6 (35)	0 (0)	**0.008**
Obesity, *n* (%)	3 (18)	2 (11)	0.66
Cardiac symptoms, *n* (%)	0 (0)	0 (0)	1.00

*BMI* body mass index

Bold indicates *p* < 0.05.

### Characteristics related to pompe disease

**Fig. 1:**
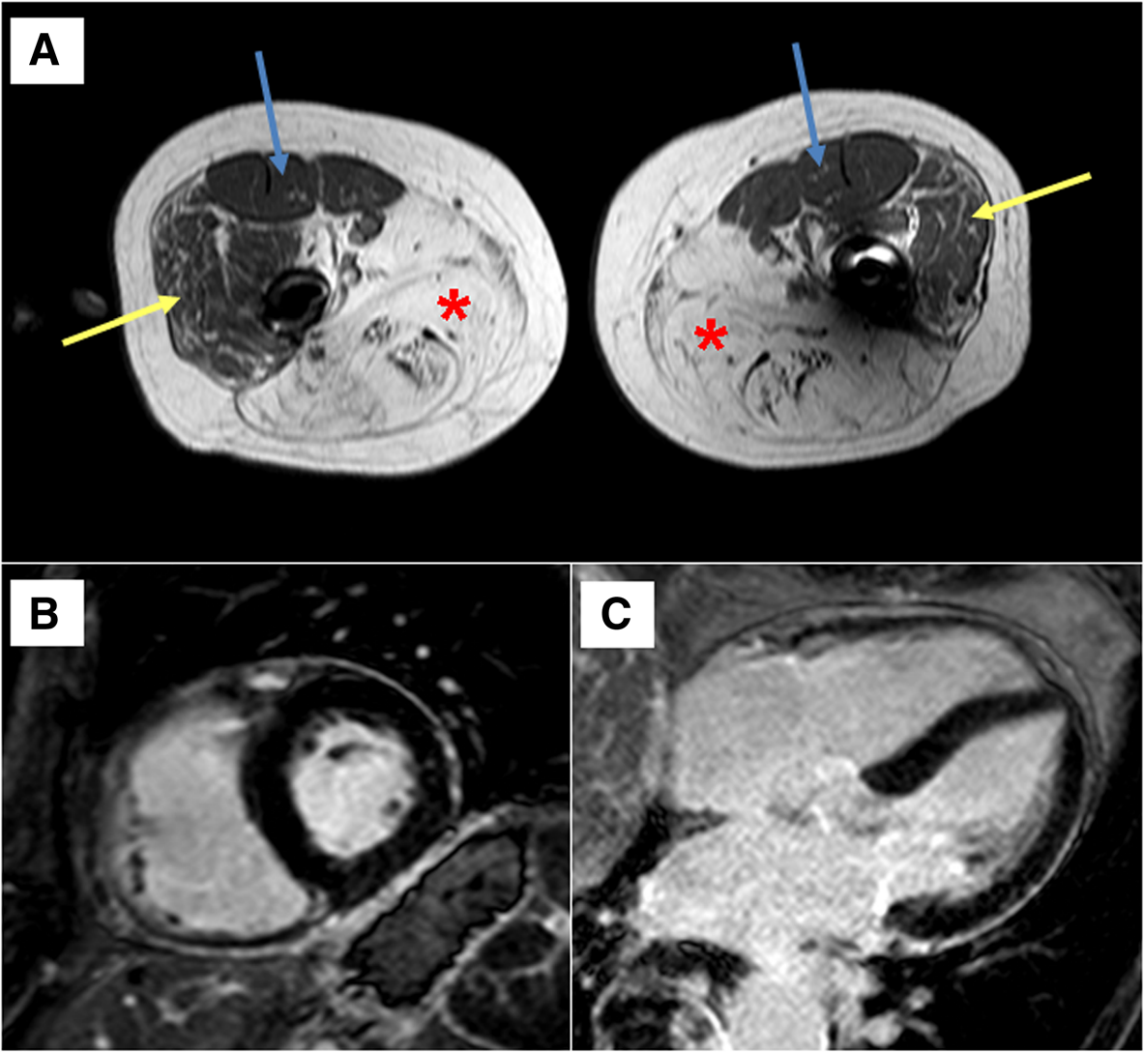
**a** Transverse T1-weighted spin-echo CMR of the thigh musculature in a patient with late-onset Pompe disease (LOPD). The adductor and ischiocrural muscles show symmetrical and confluent hyperintensity indicative of severe fibrosis and lipodystrophy (*red stars*). Some patchy fatty replacement can also be seen in the vastus lateralis muscles (*yellow arrows*) and, to a much lesser extent, in the vastus medialis muscles (*blue arrows*). **b**–**c** Short and long-axis late gadolinium enhancement (LGE) CMR images of the same patient showing no myocardial involvement (no fibrosis, no fatty replacement)

As illustrated in Table 2, patients with LOPD specified an average disease duration of 19 ± 14 years (median and IQR: 17 years; 8–29 years) with a median age at diagnosis of 30 year (20–43 years). 16 out of 17 patients showed proximal and symmetrical limb-girdle weakness (Fig. 1a). Gait abnormalities were present in all of these patients, and constant use of walking aids was necessary in three individuals. Mean walking distance in the 6 min walk test was 398.4 ± 156.5 m with a range between 164 and 610 m (*n* = 14). Upright forced vital capacity (FVC) as measured using a handheld electronic spirometer was reduced to 62.0% ± 29.2% from the predicted value. Five patients (29%) showed normal FVC, and eight individuals (47%) already received nighttime NIV prior to enrollment. Twelve patients (71%) had been receiving ERT in a standard dosage (alglucosidase alfa, 20 mg/kg body weight every two weeks) for 72 months on average (29–83 months).

**Table 2:** Pompe disease related patient characteristics

	Pompe
	*N* = 17
Age at diagnosis, years	31 ± 14
Disease duration, years	19 ± 14
Hypoventilation, *n* (%)	9 (53)
Mechanical ventilatory support, *n* (%)	8 (47)
Severely impaired FVC*, *n* (%)	7 (44)
Limb girdle weakness, *n* (%)	16 (94)
Walking aid necessary, *n* (%)	3 (18)
Six minute walk test, m	410 (234–512)
Enzyme replacement therapy, *n* (%)	12 (71)
Duration of enzyme therapy, months	72 (29–83)

* - <50% of the predicted value; FVC-upright forced vital capacity

### CMR results, conventional parameters

**Fig. 2:**
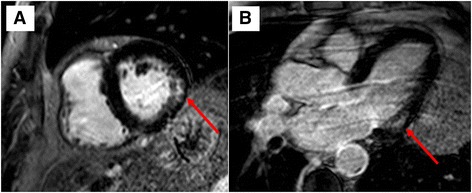
CMR of a patient with late-onset Pompe disease (LOPD) showing non-ischemic (intramural) late gadolinium enhancement (LGE) in the basal left ventricular inferolateral wall segments (*red arrows*) in short- (**a**) and long-axis views (**b**)

Data related to CMR examinations are shown in Table 3. Both patients and controls had left and right ventricular (LV/RV) dimension, including LV mass, and ejection fraction within the normal range without any significant difference between both groups. Only one patient showed mild septal hypertrophy compared to none of the controls. Eighteen percent (*n* = 3) of the patients showed LA dilatation compared to none of the controls (*p* = NS). Moreover, three patients (18%) with adult onset of disease, had LGE with a non-ischemic pattern, located mid-wall in the basal inferolateral segment (Figs. 1b-c and 2). Two of these three patients had a history of hypertension associated with LA dilatation as well as elevated cholesterol levels and an increased body weight. Interestingly, the same two (male) patients showed borderline septal thickness (12 mm). In the third (female) LGE-positive patient, we did not find any other pathological CMR finding. Interestingly, this patient was obese (BMI 30.4) and reported a very long disease duration of 34 years from the occurrence of initial symptoms.

**Table 3:** CMR parameters in the study and control groups

	Pompe	Controls	*P*-Value
	*N* = 17	*N* = 18	
Heart rate, bpm	69 ± 10	64 ± 12	0.14
BSA, kg/m^2^	0.9 ± 0.2	0.9 ± 0.2	0.90
LV-EDV index, ml/m2	75 ± 12	75 ± 15	0.85
LV-ESV index, ml/m2	28 ± 7	30 ± 7	0.40
LV-EF, %	62 ± 6	61 ± 4	0.40
LV mass index, g/m^2^	48 ± 8	53 ± 14	0.17
LV hypertrophy, *n* (%)	1 (6)	0 (0)	0.49
LV mass/volume ratio	0.65 ± 0.10	0.71 ± 0.13	0.14
RV-EDV index, ml/m2	74 ± 14	77 ± 14	0.54
RV-ESV index, ml/m2	30 ± 9	32 ± 10	0.42
RV-EF, %	60 ± 7	60 ± 9	0.73
LV-GLS, %	−19 ± 2.5	−19.1 ± 2.2	0.93
LV-GRS, %	41.3 ± 8.2	34.4 ± 6.9	**0.012**
LV-GCS, %	−20.8 ± 2.5	−18.7 ± 2.4	**0.015**
LA dilatation, *n* (%)	3 (18)	0 (0)	0.10
LGE, *n* (%)	3 (18)	0 (0)	0.10
Global ECV, %*	27.3 ± 4.5	25.4 ± 2.5	0.18
Septal ECV, %*	28.4 ± 4.9	25.9 ± 2.8	0.12
LW ECV, %*	26.2 ± 4.2	24.9 ± 2.5	0.33

*LV* left ventricle, *EDV* end-diastolic, *ESV* end-systolic, *EF* ejection fraction, *GLS* global longitudinal strain, *GRS* global radial strain, *GCS* global circumferential strain, *LA* left atrium, *LGE* late gadolinium enhancement, *ECV* extracellular volume fraction, *LW* lateral wall

*ECV measurement was performed in *N* = 14 Pompe patients and *N* = 16 controls

Bold indicates *p* < 0.05.

### CMR results-advanced imaging parameters: feature-tracking derived strain

Regarding functional (myocardial deformation) parameters (Table 3), there was no significant difference between feature-tracking derived GLS in patients and controls (−19 ± 2.5% vs. −19.1 ± 2.2%, *p* = NS). Some minor differences were measured in the Pompe group in comparison to the control group regarding GCS (−20.1 ± 2.5% vs. –18.6 ± 2.2%, *p* = 0.011) and GRS (41.3 ± 8.2% vs. 34.5 ± 6.9%, *p* = 0.012). Moreover, within the Pompe group both GCS and GRS were relatively higher in hypertensive compared to normotensive patients (−23.9 ± 1.4% vs. –20.2 ± 2.3%, *p* = 0.017 and 52.0 ± 5.4% vs. 39.0 ± 7.1%, *p* = 0.028). However, all obtained global strain values were within normal range in both groups.

### CMR results-advanced imaging parameters: ECV measurement

There was no significant difference in global ECV between Pompe patients (*n* = 14) and controls (*n* = 16) in whom successful pre- and post-contrast T1-mapping was achieved (27.3 ± 4.5% vs. 25.4 ± 2.5%, *p* = NS). The non-significant differences were unchanged when looking at septal and lateral wall (LW) ECV values. Increased global ECV values (>30%) were found in three (21%) of the Pompe patients - but in none of the controls. Among these three, two were also LGE-positive. However, the increase in ECV involved equally the septum and mid-ventricular LV lateral wall, in areas where no LGE could be visually depicted.

### Follow-up for cardiovascular events

Patients were clinically followed up for occurrence of cardiovascular events for a median of 25 months (11–29 months). During this follow-up period, one LGE-positive patient with a high cardiovascular risk profile (diabetes, hypertension, high cholesterol) experienced an acute coronary syndrome with subsequent diagnosis of two-vessel coronary artery disease, 23 months after inclusion to the study. No other cardiovascular events were registered in the above time interval.

## Discussion

To the best of our knowledge, this is the first study aiming to detect even subclinical myocardial involvement by means of comprehensive CMR, including conventional and advanced techniques — in a population of adult patients with LOPD and no previous history of cardiac disease. The major findings of this study can be summarized as follows and are in contrast to previous findings in early infantile-onset Pompe disease [3]: (i) neither conventional (cine) nor advanced (feature-tracking based strain) imaging could depict subclinical myocardial systolic dysfunction; (ii) some Pompe patients demonstrated presence of non-ischemic LGE in the basal LV inferolateral wall and showed elevated ECV suggestive of myocardial interstitial fibrosis; (iii) non-specific abnormalities such as LA dilatation were present in a few Pompe patients, while LV hypertrophy was seen only in one.

A study using state-of-the-art CMR of functional and/or structural myocardial abnormalities in LOPD was necessary since previous studies (mostly using echocardiography and a small sample size) showed rather heterogeneous and inconsistent results [5–8]. In these earlier reports, only a minority of adult Pompe patients demonstrated LV hypertrophy (always mild), whereas a small number showed mild degrees of systolic/diastolic dysfunction [5–7]. Interestingly, a more recent study found no echocardiographic abnormalities, including advanced 2D strain imaging, in 12 patients with LOPD [8]. Moreover, CMR was performed in a subgroup of their patients and no myocardial LGE was detected [8]. Unfortunately, how many patients underwent CMR and cardiovascular comorbidities are not reported.

In our present study, non-ischemic LGE was present in a small proportion of patients and its cause is debatable. A similar intramural LGE-pattern in the basal LV inferolateral wall has already been described in patients with impaired cellular energy metabolism due to genetic mitochondrial myopathy and seems to be at least partially related to a regional increase in LV wall stress [17]. However, even though mild accumulation of glycogen seems to be present also in cardiomyocytes, histopathological studies failed to depict myocardial cell death or fibrosis in LOPD [18]. On the other hand, both non-ischemic LGE and ECV increase have been described in association with hypertensive heart disease [19]. In this context, it needs to be emphasized that two of the three LGE-positive patients in the current study were hypertensive and showed a high global ECV value in the presence of a dilated LA. Yet, in contrast to our present results, ECV increase in hypertensive patients appears to be related to an increase in LV mass and a decrease in circumferential deformation parameters [19]. Thus, a clear delineation between the impact of glycogen storage and any comorbidity potentially causing focal or diffuse fibrosis may be impossible in patients with LOPD. Additionally, the minor differences in radial and circumferential deformation between Pompe and control groups can be explained by different load conditions since GCS and GRS were reported to differ significantly from controls exclusively in hypertensive patients with antihypertensive medication [20]. Finally, compared to the aforementioned study by Morris et al., our patients were older (mean age 50 year vs. 38 years), more frequently male (65% vs. 50%) and some had already comorbidities like hypertension or diabetes [8]. These factors per se might explain the occurrence of LA dilatation, suggesting impaired LV filling in the hypertensive individuals at least.

Taken together, in a small proportion of patients with LOPD mild and non-specific cardiac abnormalities can be detected by CMR and they possibly result from an interplay between glycogen storage and concomitant disease. No clear impact of the findings on short-term to mid-term prognosis was found. However, their long-term consequences remain uncertain and yet to be clarified. Nevertheless, even mild cardiac involvement is only rarely observed in patients with LOPD and does not seem to be of any prognostic value. Therefore, comprehensive CMR studies are not routinely required in these patients from a clinical point-of-view. Obviously, there are no sufficient and specific data to suggest any evidence-based recommendations for cardiac monitoring in LOPD patients. Previous reports addressing e.g. metabolic conditions or rare neuromuscular disorders suggested cardiac evaluations comprising ECG and echocardiography at diagnosis and thereafter at least every 5 years in the case of normal findings. Based on the results of this study, we would recommend a baseline cardiac assessment, including clinical history, physical examination, a 12-lead ECG and echocardiography at diagnosis. In case of normal findings, additional CMR is not required and repeated cardiac evaluations can be performed every 4–5 years. However, in case of abnormal cardiac findings using conventional methods, additional CMR may be scheduled in order to better characterize and assess the nature and clinical value of the respective findings since LOPD patients may also suffer from other (ischemic as well as non-ischemic) diseases.

Obviously, the sample size was still small in this study. However, Pompe disease is a rare condition and CMR may be challenging and sometimes impossible in patients with advanced respiratory muscle weakness. Taking this into account, the present study group of 17 patients with comprehensive CMR is noteworthy. Our control group consisted of age- and gender-matched healthy controls, while some of the Pompe patients had comorbidities potentially causing structural myocardial alterations. In addition, chronic respiratory muscle weakness itself may have a structural impact on the heart in affected patients. Echocardiography was not systematically performed at the time of CMR, so comprehensive data on diastolic function were not available. However, it is well known that the diagnosis of diastolic dysfunction by echocardiography has limited reliability, particularly in the initial stages of diastolic dysfunction [21]. Finally, a large proportion of our patients were receiving ERT which may reduce or mask any sequelae of glycogen accumulation in the myocardial tissue. Given the fact that ERT for Pompe disease is widely available in industrialized countries, we consider it unlikely that larger numbers of untreated patients will undergo CMR in the future. However, existing echocardiography-based data support that ERT has no significant impact on cardiac parameters in LOPD [6, 7].

### Conclusions

In contrast to the early infantile-onset form of Pompe disease, mild and rather non-specific cardiac abnormalities can be detected by CMR only in a small proportion of patients with late-onset Pompe disease. The observed structural abnormalities seem to result from an interplay between the storage disease and other comorbidities and they did not affect short-term to mid-term prognosis in patients with LOPD.

## Abbreviations

CK: Creatine kinase
CMR: Cardiovascular magnetic resonance
ECV: Extracellular volume
ERT: Enzyme replacement therapy
GAA: α–1,4-glucosidase
LGE: Late-gadolinium-enhancement
LOPD: Late-onset pompe disease
LV: Left ventricle
LV-EDV: Left ventricular end-diastolic volume
LV-EF: Left ventricular ejection fraction
LV-ESV: Left ventricular end-systolic volume
MOLLI: Modified look-locker inversion recovery
NIV: Non-invasive ventilation
RV: Right ventricle
RV-EDV: Right ventricular end-diastolic volume
SSFP: Steady-state-free-precession
